# Medical Cannabis for Older Patients—Treatment Protocol and Initial Results

**DOI:** 10.3390/jcm8111819

**Published:** 2019-11-01

**Authors:** Ran Abuhasira, Addie Ron, Inbal Sikorin, Victor Novack

**Affiliations:** 1 Cannabis Clinical Research Institute, Soroka University Medical Center and Faculty of Health Sciences, Ben-Gurion University of the Negev, Be’er-Sheva 8457108, Israel; ranabu@post.bgu.ac.il; 2 NiaMedic Healthcare and Research Services, Bnei-Brak 5126107, Israel; addie@niamedic.com (A.R.); inbal@niamedic.com (I.S.)

**Keywords:** older adults, medical cannabis, marijuana, protocol

## Abstract

Older adults may benefit from cannabis treatment for various symptoms such as chronic pain, sleep difficulties, and others, that are not adequately controlled with evidence-based therapies. However, currently, there is a dearth of evidence about the efficacy and safety of cannabis treatment for these patients. This article aims to present a pragmatic treatment protocol for medical cannabis in older adults. We followed consecutive patients above 65 years of age prospectively who were treated with medical cannabis from April 2017 to October 2018. The outcomes included treatment adherence, global assessment of efficacy and adverse events after six months of treatment. During the study period, 184 patients began cannabis treatment, 63.6% were female, and the mean age was 81.2 ± 7.5 years (median age-82). After six months of treatment, 58.1% were still using cannabis. Of these patients, 33.6% reported adverse events, the most common of which were dizziness (12.1%) and sleepiness and fatigue (11.2%). Of the respondents, 84.8% reported some degree of improvement in their general condition. Special caution is warranted in older adults due to polypharmacy, pharmacokinetic changes, nervous system impairment, and increased cardiovascular risk. Medical cannabis should still be considered carefully and individually for each patient after a risk-benefit analysis and followed by frequent monitoring for efficacy and adverse events.

## 1. Introduction

The recent interest and use of medical cannabis (MC) are growing substantially in many countries. The regulations on its use vary among countries, affecting medical practice and experience [1]. Current public opinion is that cannabis has the therapeutic potential to treat and cure a long list of diseases, but there is a large gap between that opinion and the current evidence in the medical literature [2]. Another common opinion is that MC is mainly used by young adults. However, the use of MC by older adults is increasing [3], and studies show variable prevalence, ranging from approximately 7% to more than one-third, depending on the country [4,5]. Recreational use of cannabis by older adults is also increasing substantially, especially in the United States [6].

Relief of suffering and promotion of functional status and quality of life are major goals of geriatric medicine. Chronic pain, Parkinson’s disease, depression, sleeping disorders, and malnutrition are all common among older adults [7,8,9,10,11,12]. Current medical treatments of these syndromes can have serious adverse events, frequently endangering patients’ health. For example, some non-steroidal anti-inflammatory drugs (NSAIDs) are associated with gastrointestinal bleeding, renal impairment, and cardiovascular adverse events [13]. Sedative hypnotics can cause psychomotor impairment, dizziness, confusion, increased risk of falls, next-day somnolence, impairment of driving skills, orthostatic hypotension, and blood electrolyte impairment [14]. Opioid treatment causes constipation, nausea, vomiting, drowsiness, delirium, sedation, anticholinergic effects, falls, and respiratory depression, which is the most serious potential adverse effect [13]. Beyond individual factors, current concerns about opioid-related deaths have greatly influenced our thinking about pain management and medication treatment [15].

### 1.1. Efficacy and Indications for Medical Cannabis in Older Adults

The geriatric population may benefit from cannabis treatment for a variety of symptoms, such as chronic pain, sleep difficulties, tremor, spasticity, agitation, nausea, vomiting, and reduced appetite. Cannabis may also be useful in palliative care. However, currently, there is a dearth of evidence about the efficacy of cannabis in older adults for any of these symptoms. This has been emphasized in several reviews [16,17,18] and in large reports such as the report of the National Academies of Sciences in the United States [19] and the Information for Health Care Professionals in Canada [20].

### 1.2. Chronic Pain

Chronic pain is one of the most common indications for prescribing MC. The report by the National Academies of Sciences concludes that cannabis is effective for the treatment of chronic pain in adults [19]. Despite this conclusion and a large number of studies, including randomized controlled trials, the efficacy for cannabis as a chronic pain medication remains in dispute [21]. Pain relief is very often cited as a reason for MC use among older individuals. For example, 89.7% of the older patients in the Colorado MC registry listed pain as their primary or secondary condition [4]. All the large studies that evaluated cannabis for pain have included older adults in the inclusion criteria, but their number was small, or they were not analyzed separately for safety and efficacy [21,22]. 

### 1.3. Parkinson’s Disease

Parkinson’s disease (PD) is a common neurodegenerative disease found mostly among older adults, which is caused by dopaminergic neuron loss. It is mainly characterized by motor symptoms that include bradykinesia in combination with resting tremor or rigidity [23]. PD also has a distinct prodromal stage identified by non-motor symptoms, such as olfactory dysfunction, constipation, urinary dysfunction, depression, anxiety, and pain [24]. Two small-scaled randomized controlled trials failed to demonstrate the efficacy of cannabis in treating the motor symptoms of PD [25,26]. However, cannabis might improve quality of life in PD and relieve other non-motor symptoms [27].

### 1.4. Sleep Difficulties

Approximately 50% of people above age 65 complain about sleeping difficulties, and there is an increase in sleep disturbances in old age [28]. Care must be taken not to mistake geriatric sleep complaints for physiological aging, as these complaints are mainly attributable to medical, psychiatric and health-related burdens [29]. It should be noted that sleep disturbances are among the most frequent complaints of cannabis withdrawal, and are a major cause for continued use after attempts to quit [30]. Both pharmacological and non-pharmacological treatments are used to address sleep disorders among older individuals [31]. A meta-analysis evaluating the therapeutic effect of cannabis on sleeping disturbances has not reached a decisive conclusion. The effects of cannabis on the sleep–wake cycle are also unclear [32], though some research suggests that cannabis might aid in sleep disorders due to its anxiolytic effect [30].

### 1.5. Nausea and Vomiting

A Cochrane review concluded that “Cannabis-based medications may be useful for treating refractory chemotherapy-induced nausea and vomiting” [33]. A more recent review states that there is low-quality evidence that cannabinoids prevent nausea and vomiting as compared to other agents or a placebo [34]. The only study that addressed this issue in older adults was in 1982, and it found no difference between tetrahydrocannabinol (THC) and prochlorperazine in reducing nausea and vomiting [35].

### 1.6. Post-Traumatic Stress Disorder (PTSD)

The efficacy of cannabis treatment for PTSD in older individuals was not evaluated thus far in any study. Several studies evaluated the efficacy of cannabis treatment for PTSD in younger adults, but these studies also failed to demonstrate a clear effect of MC treatment for these patients [21].

### 1.7. Dementia

Dementia is a prevalent condition in older adults causing cognitive decline [36]. Small studies that used Dronabinol, oral synthetic Δ^9^-THC, or an extract of THC from plants, showed it improved neuropsychiatric symptoms, agitation, nocturnal motor activity, sleep duration, and meals consumption in dementia patients, while only a few serious adverse events were observed [37,38,39].

However, a study conducted with Namisol, an oral tablet containing ≥98% natural ∆^9^-THC, showed it did not reduce neuropsychiatric symptoms, agitation, activities of daily living, or improved quality of life in dementia patients [40]. 

### 1.8. Palliative Treatment

A recent systematic review and meta-analysis were unable to make any recommendation about the use of cannabis in palliative care after evaluating studies that included mainly younger adults and a small number of older adults [41].

## 2. Special Considerations and Precautions

### 2.1. Pharmacokinetics, Pharmacodynamics, and Drug Interactions

It is well known that aging is associated with substantial changes in pharmacokinetics and pharmacodynamics. For instance, hepatic drug clearance, as well as renal elimination, are both decreased in older adults. Furthermore, aging is associated with increased body fat and decreased lean body mass [42], which increases the volume of distribution for lipophilic drugs, such as cannabis. Two small studies evaluated the pharmacokinetics and pharmacodynamics of older adults who received an oral drug containing pure THC. These phase I and phase II trials included 12 healthy older adults and 10 older adults with dementia, respectively, and found smaller pharmacodynamic effects of THC in both groups, although the pharmacokinetic data showed substantial inter-individual variation [43,44]. Interaction between cannabis products and other drugs is also largely unknown, as the current evidence from human studies is sparse [45]. Concomitant administration of cannabis with other drugs that influence the hepatic CYP family enzymes may greatly alter the metabolism of the cannabinoids [46]. This issue is especially important in the geriatric population, where polypharmacy is common [47].

### 2.2. Nervous System Impairment

The common adverse effects experienced by patients due to cannabis use include dizziness, euphoria, drowsiness, confusion, and disorientation [16]. These effects are particularly important in the geriatric population, which may have conditions such as dementia, frequent falls, mobility problems, hearing, or vision impairments [48]. The long-term effect of adult cannabis use on cognition is unclear. Two systematic reviews showed evidence that long-term use of cannabis is associated with negative effects on some cognitive functions, but evidence of enduring negative effects was weak [49,50]. 

### 2.3. Cardiovascular Risks

The effects of cannabis on cardiovascular diseases are not yet well established. In recent years, however, there has been an increasing number of case series and reports concerning young, healthy recreational cannabis users who suffer from arrhythmias, myocardial infarction, and even sudden cardiac death [51]. Direct causality has not been proven, but the implication is that care must be taken concerning older adults since they have more cardiovascular comorbidities and risk factors. 

The acute cardiovascular effects of cannabis, based on studies performed on younger individuals, include an increase in sympathetic activity that causes an increase in heart rate, cardiac output, and myocardial oxygen demand. Tolerance of the effects of cannabis on heart rate develops rather quickly in young people [52].

This article aims to present a novel medical cannabis treatment protocol in older adults and the initial results from its use. The protocol will be presented in the Discussion segment of the manuscript.

## 3. Methods

### 3.1. Patients and Methods

Israeli medical cannabis regulations include a number of indications and recommendations for its use [1]. We have adopted the general recommendations to suit the physiological and pathophysiological needs of the elderly. In 2017, NiaMedic established a specialized geriatric clinic to provide MC therapy within a comprehensive geriatric platform. We have followed 184 consecutive patients above 65 years of age prospectively who were treated with MC from April 2017 to October 2018. The patients were followed for at least six months since treatment initiation. The inclusion criteria were age of 65 years and above and any of the following indications for cannabis treatment: chronic cancer pain and non-cancer pain, Parkinson’s disease, sleep disorders, anorexia, post-traumatic stress disorder, spasticity, and palliative treatment. The exclusion criteria were severe cardiovascular diseases, such as heart failure or a recent major myocardial infarction, liver failure, psychotic comorbidities, and those with a history of addictions. The follow-up evaluation includes detailed questioning regarding adverse events, adherence to treatment, and its efficacy.

### 3.2. The Treatment Protocol

As previously mentioned, the regulations of cannabis and its products vary by country, which affects the clinical experience of physicians. In Israel, cannabis can be prescribed for the following conditions: nausea and vomiting due to chemotherapy treatment, cancer-associated pain; Crohn’s disease, ulcerative colitis; neuropathic pain; AIDS patients with Cachexia; multiple sclerosis, Parkinson’s disease, Tourette syndrome, epilepsy (both adult and pediatric population); palliative treatment; post-traumatic stress disorder [1]. The initially approved dosing is 20 grams of cannabis compound per month (0.6 grams per day), with a cannabis product that contains the lowest concentration of active ingredients, but without limitation to the ratio of the different cannabinoids. The only cannabinoid-based medicine that is approved at the time of this manuscript preparation is Nabiximols, and its use is infrequent. Thus, we provide here our approach that is based on the available literature, data analysis, and our clinical experience with treating older adults with herbal cannabis, which includes the cohort above and previously published data [53]. We offer many recommendations consistent with Minerbi et al. and MacCallum et al. [17,54].

### 3.3. Ethics

Our study collected all the relevant clinical data as a part of the routine medical practice. Thus, Soroka University Medical Center institutional review board (IRB) Committee approved the protocol and waived the request for informed consent (confirmation number 0036-18-SOR). All clinical investigations were conducted according to the principles expressed in the Declaration of Helsinki.

## 4. Results

**Figure 1:**
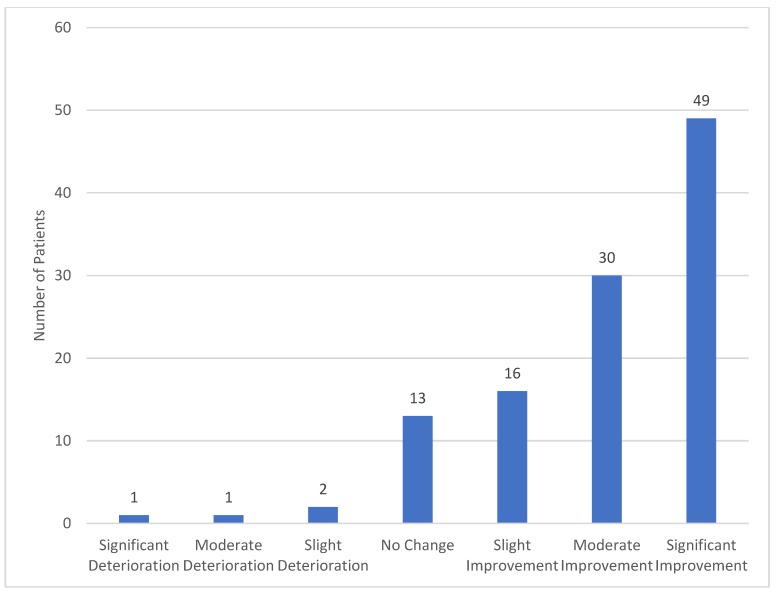
Global assessment of the general effect of cannabis on the patient’s condition after six months of treatment (*n* = 112).

We present here initial data from a cohort of patients who initiated MC therapy between April 2017 and October 2018. Most of our patients, 83.2% (*n* = 153) were 75 years of age or older, and 63.6% (*n* = 117) were females. The demographic characteristics, the comorbidities of the patients, and the indications for cannabis treatment are detailed in Table 1. When we evaluated the patients after six months of MC treatment, we found that 58.1% were still using cannabis, 8.1% discontinued the treatment, 10.9% were lost to follow-up, and 17.9% did not complete six months of treatment by the time of the analysis. Of the 122 patients eligible to respond, 91.8% (*n* = 112) globally assessed the effect of cannabis on their general condition, with 84.8% of them reporting some degree of improvement (Figure 1). Of the patients who were still treated with cannabis, 33.6% reported adverse events, the most common of which were dizziness (12.1%), sleepiness and fatigue (11.2%), dry mouth (5.6%), and psychoactive sensation (5.6%). Since well-established and evaluated protocols for treatment of older adults with cannabis do not exist, we have developed our own approach based on close follow-up of effects, adverse events, and slow titration.

## 5. Discussion

Our results show that cannabis was well tolerated by most of our patients due to a fairly high adherence to treatment after six months with a relatively low number of adverse events, and specifically, serious adverse events. Most of the patients were satisfied with the treatment and believed it was beneficial to their general health. These results are similar to the results of a previous study performed by our group [53], and to other studies performed in the United States [55,56]. However, the treatment is not suitable for all patients, and its use should be considered after failure of evidence-based treatments. We believe that the primary focus should be on the safety of the treatment—primum non nocere. Slow titration and frequent monitoring are the cornerstones of cannabis treatment for older adults. The efficacy of the treatment should be evaluated according to different indications in clinical trials and registry studies. We call for the implementation of our protocol in clinical practice to evaluate the benefit of cannabis treatment.

### 5.1. Patient Selection

**Figure 2:**
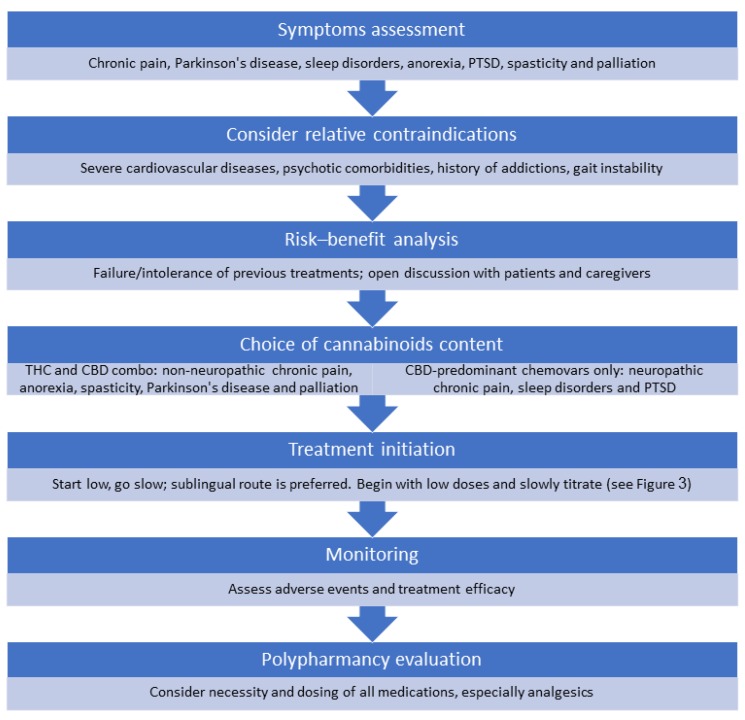
Practical approach to cannabis treatment in older adults. PTSD—post-traumatic stress disorder, THC—tetrahydrocannabinol, CBD—cannabidiol.

We begin with an evaluation of symptoms and the possible potential of cannabis to alleviate these symptoms (Figure 2). We distinguished among the categories of chronic cancer pain and non-cancer pain management, Parkinson’s disease, sleep disorders, anorexia, post-traumatic stress disorder, spasticity, and palliative treatment. As noted above, the evidence for the efficacy of cannabis in treating the indications above is sparse, especially concerning older adults. Thus, cannabis is usually not the first line of treatment, and its use should be considered experimental for most cases.

There are no absolute contraindications for cannabis treatment in the geriatric population, but physicians must consider the risks of the treatment. We suggest avoiding treatment in patients with severe cardiovascular diseases, such as heart failure or a recent major myocardial infarction, psychotic comorbidities, and those with a history of addictions. Caution is also needed for patients with gait instability and nervous system impairment, polypharmacy, and reduced drug elimination mechanisms.

### 5.2. Selection of The Compound

Herbal cannabis can be classified in different ways [57], and the most common classification method in medical use is according to the composition of the main known cannabinoids—THC and cannabidiol (CBD). The cannabis plant also contains other cannabinoids, as well as terpenes and flavonoids. The effects of the latter two are currently under-researched and thus less recognized than THC and CBD [58]. Most cannabis chemovars (“strains”) are divided between THC-predominant, containing between 10% to 20% THC and less than 5% of CBD, and CBD-predominant, containing 10% to 24% CBD and less than 5% of THC. The content of other cannabinoids or other active components is not always reported and/or verified. Current cannabis research and manufacturing enable us to discuss cannabis medications only at the level of these cannabinoids, THC and CBD.

Regardless of the chemovar and cannabinoid content chosen, we act by the simple aphorism—“Start low, go slow, and stay as low as possible.” We prefer to choose a combination of THC-predominant chemovars and CBD-predominant chemovars for the following symptoms: non-neuropathic chronic pain, anorexia, spasticity, Parkinson’s disease, and most cases of palliative treatment. CBD-predominant chemovars only are chosen for chronic neuropathic pain, sleep disorders, and post-traumatic stress disorder. It should be noted that for chronic neuropathic pain, despite initiation of treatment with CBD-predominant chemovars, THC is sometimes added slowly to increase analgesia.

**Figure 3:**
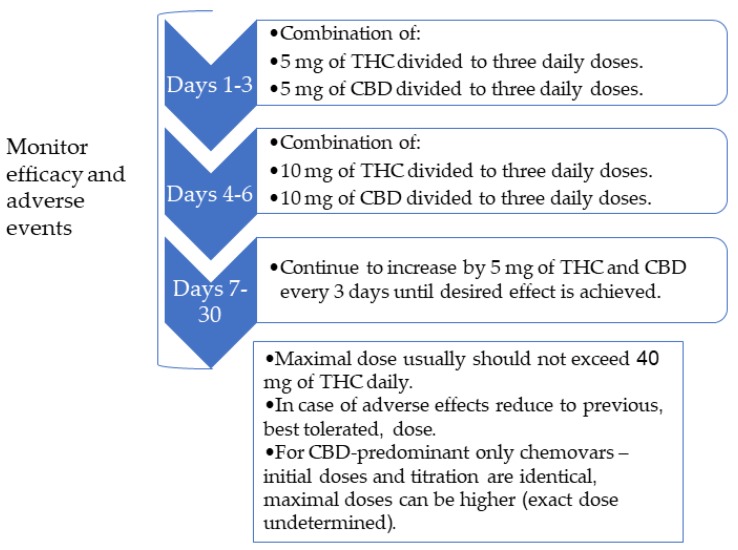
Titration algorithm to initiate herbal cannabis treatment. THC—tetrahydrocannabinol, CBD—cannabidiol.

The common therapeutic doses for most indications are 5–30 mg of THC and CBD per day divided to two-three doses, depending on the indication, the symptoms, and the current medication regimen. We usually start with 5 mg of THC and CBD a day divided into three doses (approximately 1.7 mg in a single dose) with an upward titration of 5 mg every three days (Figure 3). CBD-predominant chemovars are added to THC therapy since CBD can balance the psychoactive adverse effect of THC, especially during the daytime. Slow upward dose titration of THC may promote tolerance of psychoactive adverse effects. THC can cause a euphoric effect, but reaching this effect is not required for symptom control. In cases that we choose to use a CBD-predominant chemovar only, the dosing also starts with 5 mg CBD a day divided into three doses, but the therapeutic dose might be higher eventually.

### 5.3. Selection of The Delivery Method

Smoking cannabis should be deferred due to the deleterious effects of chronic cannabis smoking on lung function and respiratory symptoms [59]. In general, the preferred method for medical cannabis administration in older adults is the sub-lingual route. The sub-lingual method of administration utilizes the epithelial cells lining the tissue underneath the tongue and avoids first-pass metabolism by the liver. This exposes the patient to higher concentrations of cannabinoids compared to oral swallowing [60], but it is less rapid than the inhaled route. A more important feature of sub-lingual administration is that it allows for more accurate control of the delivered dose since the technique is not as complicated as inhalation, and the active ingredients are not subjected to high variability in absorbance as in the oral administration [61]. For chronic conditions and symptoms, sub-lingual preparations are the mainstay of treatment with inhaled/vaporized cannabis as an add-on PRN for episodic exacerbations of symptoms. The main delivery method we use for sublingual preparation is cannabis oil extracted from whole herbal cannabis with different compositions of cannabinoids, as discussed earlier.

Inhaling cannabis delivers a high dose in a very short period, but soon thereafter, there is a relatively rapid decline [62], and abrupt changes in blood concentration of cannabinoids can increase the risk of adverse events. Inhaled cannabis is also influenced by parameters such as puff duration, volume inhaled, and time spent holding breath—all of which can substantially alter the dose administered [61].

In the oral administration route, the variability in blood concentrations among subjects is very high. Blood concentrations are lower in about 5- to 25-fold as compared to intravenous administration due to the first-pass metabolism in the intestines and liver. The peak concentration is observed only 1–2 h after administration [63]. This variability among subjects makes it difficult to achieve stable concentrations, and thus puts older adults at high risk for adverse events. Therefore, we do not recommend using oral preparations in older adults.

### 5.4. Addressing Patients Concerns 

In some instances, the stigma associated with the use of the “drug” can prevent our patients from accepting the use of medical cannabis—a relatively safe and potentially efficacious medication. Therefore, cannabis differs from other drugs in terms of public relations and the strong opinions held by many patients. Some patients dread it, while others may view it as the ultimate solution for most of their symptoms. Therefore, the explanation of the potential benefits and risks before the initiation of cannabis treatment takes on heightened importance for patients and their caregivers. This explanation should be performed by a nurse or a physician that are proficient in cannabis treatment.

### 5.5. Follow-Up

Once MC treatment is initiated and the therapeutic dose is achieved, we recommend at least monthly follow-up at first to assess adverse events and treatment efficacy. If treatment is effective and well-tolerated, consideration can then be given to revising the current concomitant drug regimen, especially with respect to the use and dosage of opioids, benzodiazepines, and other psychotropic or analgesic medications. Our experience shows that cannabis has the potential to lead to a reduction in the use of these medications [53].

## 6. Conclusions

The evidence for the efficacy and safety of cannabis in treating older adults is sparse. Nevertheless, the changing regulations, and media and public opinion require physicians to address cannabis treatment differently and offer it for suitable conditions. Potential indications for MC use in older adults include pain, sleep disturbances, nausea and vomiting, Parkinson’s disease, post-traumatic stress disorder, dementia, and palliation. The potential risks of cannabis should not be disregarded, and the emphasis needs to be polypharmacy, pharmacokinetic changes, nervous system impairment, and increased cardiovascular risk. After individual consideration and a personal risk–benefit analysis for each patient, MC treatment should be initiated slowly and gradually. Following treatment initiation, patients must be monitored frequently for adverse events and efficacy.

## Figures and Tables

**Table 1:** Demographic characteristics, indication for cannabis treatment, and comorbidities of the patients treated with medical cannabis.

Variable	Number of Patients (*n* = 184)
Demographic characteristics	
Age (years)	65–74–31 (16.8%)
	75–84–91 (49.5%)
	≥85–62 (33.7%)
Female	117 (63.6%)
Family status	Married–64 (34.8%)
	Widowed–42 (22.8%)
	Divorced–7 (3.8%)
	Unknown–71 (38.6%)
Comorbidities (*n*, %)	
Hypertension	51 (27.7%)
Dyslipidemia	30 (16.3%)
Diabetes Mellitus	25 (13.6%)
Hypothyroidism	19 (10.3%)
Osteoporosis	17 (9.2%)
Spinal Stenosis	12 (6.5%)
Obesity	10 (5.4%)
Stroke or transient ischemic attack history	9 (4.9%)
Ischemic heart disease	9 (4.9%)
Atrial fibrillation	9 (4.9%)
Parkinson’s disease	8 (4.3%)
Malignancy (different types)	7 (3.8%)
Depression	7 (3.8%)
Dementia	6 (3.3%)
Cannabis treatment indications (*n*, %)	
Non-specific chronic pain (including neuropathic pain)	105 (57.1%)
Parkinson’s disease	12 (6.5%)
Orthopedic pain	10 (5.4%)
Oncologic treatment	7 (3.8%)
Dementia	5 (2.7%)
Arthritis	5 (2.7%)
Restlessness	3 (1.6%)
Fibromyalgia	2 (1.1%)
Palliative treatment	2 (1.1%)
Others	33 (17.9%)
